# The LRP1-SHP2 pathway regulates TRPV1 sensitivity in the peripheral nervous system: Insights from amyloid beta 1–42 modulation

**DOI:** 10.1016/j.jare.2025.03.005

**Published:** 2025-03-05

**Authors:** Sung-Min Hwang, Jueun Roh, Eun Jin Go, Jing-Ying Pan, Jaeik Park, Mahbubur Rahman, YunJae Jung, Sun-Ho Lee, Inbo Han, Gehoon Chung, Sang Hoon Lee, Temugin Berta, Chul-Kyu Park, Yong Ho Kim

**Affiliations:** a Gachon Pain Center and Department of Physiology, College of Medicine, Gachon University, Incheon 21999, Republic of Korea; b Department of Histology and Embryology, Medical School of Nantong University, Nantong, Jiangsu Province 226001, China; c Department of Health Sciences and Technology, GAIHST, Gachon University, Incheon 21999, Republic of Korea; d Department of Neurosurgery, Spine Tumor Center, Samsung Medical Center, Sungkyunkwan University School of Medicine, Seoul 06351, Republic of Korea; e Department of Neurosurgery, School of Medicine, CHA University, CHA Bundang Medical Center, Seongnam-si 13496, Republic of Korea; f Department of Oral Physiology, School of Dentistry, Seoul National University, Seoul 08826, Republic of Korea; g Pain Research Center, Department of Anesthesiology, University of Cincinnati Medical Center, Cincinnati, OH, USA

**Keywords:** Amyloid beta, α2-macroglobulin, Peripheral nervous system, Transient receptor potential vanilloid 1, Low-density lipoprotein receptor-related protein 1, Src homology-2 domain-containing protein tyrosine phosphatase-2

## Abstract

**Introduction:**

Mature adults often exhibit higher pain thresholds than younger individuals. However, this phenomenon is poorly understood, especially with regards to peripheral nervous system signaling.

**Objectives:**

We investigated the involvement of amyloid beta (Aβ) in regulating heat pain sensitivity within the dorsal root ganglion (DRG) during adult maturation.

**Methods:**

We employed various in vivo and in vitro techniques to investigate the modulatory effect of Aβ_1–42_. Heat pain sensitivity alteration was examined in spared nerve injury (SNI) models of young and mature adult Aβ_1–42_-treated mice. Phosphorylation and receptor inhibition assays were performed to elucidate the molecular mechanisms involved in pathway interactions in vitro.

**Results:**

Mature adult mice had higher thermal pain thresholds and elevated levels of Aβ_1–42_ compared to younger mice*.* In vitro analyses indicated that Aβ_1–42_-induced activation of low-density lipoprotein receptor-related protein 1 (LRP1) led to phosphorylation of the src-homology domain-2–containing protein tyrosine phosphatase 2 (SHP2), which in turn inhibited transient receptor potential vanilloid 1 (TRPV1) function in primary DRG neurons. Similar mechanisms were observed in Aβ_1–42_-treated human DRG neurons. Additionally, α_2_-macroglobulin (α_2_M), a potent LRP1 agonist, also inhibited TRPV1 activity and reduced heat pain sensitivity through the LRP1-SHP2 pathway. In vivo studies with the mouse SNI model demonstrated that intraplantar injection of Aβ_1–42_ and α_2_M enhanced the paw withdrawal latency; these effects were reversed by low-density lipoprotein receptor-related protein-associated protein 1.

**Conclusion:**

The findings suggest a crucial role of Aβ in modulating heat pain sensitivity during maturation through TRPV1 inhibition. The study offers new insights into the regulation of pain sensitivity during the maturation process by revealing a novel intrinsic mechanism involving Aβ_1–42_ in heat pain sensitivity and its regulation through the LRP1/SHP2 pathway in mature adults. This pathway could be a potential therapeutic target for age-related chronic pain management.

## Highlights

• Middle-aged mice show substantially higher heat pain thresholds than younger mice.
• Middle-aged mice with raised Aβ1–42 levels have increased pain thresholds via LRP1.
• α2-Macroglobulin, a potent LRP1 agonist, notably reduces heat pain sensitivity.
• LRP1 blockade reverses the enhanced pain thresholds induced by high Aβ1–42 levels.
• LRP1-SHP interaction modulates TRPV1, impacting neural pain sensitivity.

## Graphical abstract

**Figure:**
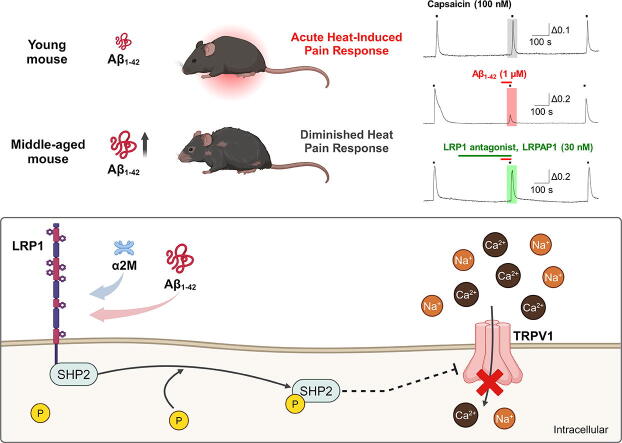

## Introduction

Pain perception varies across the lifespan, with mature adults often exhibiting higher pain thresholds compared to younger individuals. Studies have shown that older adults experience increased pressure pain thresholds, reflecting reduced sensitivity to mechanical pain stimuli [1]. Similarly, diminished responses to thermal stimuli have been observed, indicating a reduction in heat pain sensitivity with age [2]. While these findings emphasize the importance of understanding age-related differences in pain perception, the mechanisms underlying this phenomenon remain poorly understood [3,4]. Investigating these mechanisms is crucial, as they hold the potential to uncover novel strategies for pain modulation.

Emerging evidence suggests that amyloid beta (Aβ), particularly Aβ_1–42_, is a key regulator of sensory pathways. Commonly associated with neurodegenerative diseases, Aβ_1–42_ has also been implicated in neuroprotective functions and pain modulation, particularly during advanced developmental stages [5,6]. Its presence in dorsal root ganglion (DRG) sensory neurons within the peripheral nervous system (PNS) suggests that it plays a role in maintaining neuronal survival and influencing nociceptive processes [7]. However, the mechanisms by which Aβ_1–42_ modulates pain perception, especially in mature adults, remain unclear.

Low-density lipoprotein (LDL) receptor-related protein 1 (LRP1), a multifunctional receptor ubiquitously expressed in neurons [8,9], has emerged as a potential mediator of the effects of Aβ_1–42_. LRP1 plays a key role in endocytosis and intracellular signaling, facilitating the uptake and trafficking of various ligands such as apolipoprotein E, α2-macroglobulin (α2M), and Aβ [[9], [10], [11], [12], [13], [14]]. Through interactions with intracellular adaptors and scaffolding proteins, LRP1 mediates signaling cascades involving Src homology 2 (SH2) domain-containing protein tyrosine phosphatase (SHP2), which is a phosphatase that regulates signal transduction and cellular processes. The role of SHP2 in dephosphorylating TRPV1, a critical pain receptor, highlights its importance in modulating pain sensitivity [9,13,15]. Moreover, LRP1 has been identified as a modulator of neuropathic pain. Intrathecal or intracerebroventricular administration of Aβ has been shown to influence pain perception in various animal models [5,[16], [17], [18], [19]], suggesting its relevance in pain-related signaling.

In addition to the established role of LRP1 in neuropathic pain modulation, its potential interaction with key pain-sensing receptors, such as transient receptor potential vanilloid 1 (TRPV1), offers valuable insight into the molecular mechanisms underlying pain regulation. TRPV1, a critical thermo-TRP channel, plays a central role in detecting heat pain and is primarily localized in small- and medium-sized DRG neurons of the PNS. Activated by capsaicin and noxious heat stimuli, TRPV1 is integral to the mechanisms of heat hyperalgesia and other pain modalities [[20], [21], [22], [23]]. Studies in knockout mice (VR1−/−) have underscored its pivotal role in thermal hyperalgesia, further emphasizing its importance in mediating pain responses to noxious heat [[23], [24], [25], [26], [27]]. The functionality of this channel is intricately regulated by phosphorylation at multiple kinase-specific sites, which highlights its complexity in pain signaling pathways [22,[28], [29], [30]]. The delicate balance between phosphorylated and dephosphorylated states of TRPV1 is crucial for its nociceptive function [29,30]. Additionally, protein tyrosine phosphatases, such as SHP-1, modulate TRPV1 activity through dephosphorylation [13,[31], [32], [33], [34]].

Despite extensive research, the precise mechanisms through which Aβ_1–42_ modulates pain sensitivity in the PNS remain poorly understood. Therefore, this study aimed to investigate the role and underlying mechanisms of Aβ_1–42_ in modulating heat pain sensation across developmental stages, focusing on young and mature adult mice. We hypothesized that the analgesic effects of Aβ_1–42_ would be mediated by its regulation of TRPV1 function, particularly in the context of heat hyperalgesia. This study revealed a novel regulatory mechanism wherein Aβ_1–42_ modulates TRPV1-mediated heat pain via the endogenous LRP1-SHP2 signaling pathway in the PNS. These insights not only deepen our understanding of pain regulation in mature adults, but also highlight a promising therapeutic target for managing chronic pain.

## Materials and methods

### Animals and treatment

All surgical and experimental procedures were reviewed and approved by the Institutional Animal Care and Use Committee of the College of Medicine at [BLINDED FOR REVIEW] (approval number: LCDI-2020–0011; approval date: May 23, 2020). Young and mature adult male C57BL/6N mice were purchased from Orientbio (Sungnam, Korea). LRP1^flox/flox^ mice (RRID: IMSR_JAX:012604) were purchased from the Jackson Laboratory (Bar Harbor, ME, USA). TRPV1-cre; LRP1^flox/flox^ mice were generated by intrathecal administration of 2.0 × 10^12^ GC/mL adeno-associated virus serotype 5 (AAV5)-mTRPV1-Cre-T2A-tdTomato-polyA or AAV5-mTRPV1-tdTomato at 25 days after birth. Four weeks after the injection, the conditional knockout mice were used for multiple behavioral studies, with each study conducted at least 3 days apart using the same mice. The mice were allowed to acclimatize to the facility (12 h light/12 h dark cycle) for at least 1 week before the experimental procedures.

### Reagents

Aβ_1–42_ and low-density lipoprotein receptor-related protein-associated protein 1 (LRPAP1) were purchased from Abcam (Cambridge, UK). Aβ_1–42_ was dissolved in the appropriate vehicles according to the lot number of the stock, and LRPAP1 was dissolved in UltraPure DNase/RNase-Free Distilled Water (Gibco, Waltham, MA, USA). Capsaicin and α_2_M were purchased from Sigma-Aldrich (St. Louis, MO, USA), and the stock solution was prepared with 99.5 % ethanol. NSC-87877, PTPiIII, and SHP099 were purchased from Cayman (Ann Arbor, MI, USA), and the stock solutions were prepared with dimethyl sulfoxide. All stock solutions were stored at − 20 °C except for LRPAP1 and PTPiIII solutions, which were stored at − 80 °C.

### Mouse pain model, drug delivery, and intrathecal injection

To induce spared nerve injury (SNI) in mice, the left common peroneal and tibial nerves were cut under isoflurane anesthesia. To evoke the spontaneous nociceptive response, 20 μL of 0.125 μg capsaicin with the vehicle or drugs (Aβ_1–42_ [1 μg], α_2_M [10 nM], LRPAP1 [0.5 μg], and SHP099 [2 μg]) were injected into the plantar surface of the hind paw. The spontaneous pain nociceptive behavior was evaluated by counting the time (s) of licking, lifting, and shaking the injected paw every min for 5 min [35,36]. Twenty microliters of the vehicle or drugs (Aβ_1–42_ [1 μg], α_2_M [10 nM], LRPAP1 [0.5 μg], and SHP099 [2 μg]) were delivered using the intrathecal route. The intrathecal injection was administered using a 30 G needle between L5 and L6.

### Behavior test

Animals were acclimatized to the environment at least 2 days before testing. Thermal sensitivity and heat hyperalgesia were tested using the hot plate and Hargreaves tests, respectively [37,38]. The hot plate test was conducted to assess the voluntary movement of individual mice placed within a chamber. The recorded data are presented as the time (s) taken for the mouse to withdraw its paw from the plate, with a cutoff of 60 s. The Hargreaves test was performed by measuring the paw withdrawal latency (PWL) against the radiant heat. The basal PWL was set to 9–12 s, with a cutoff of 20 s to prevent tissue damage. The von Frey test was performed to test mechanical allodynia. For this test, the mice were confined in transparent acrylic boxes placed on a metal mesh floor away from the floor, and the hind paws were stimulated with several von Frey hairs with logarithmically increasing stiffness (0.02–2.56 g). Dixon’s up-down method was used to determine the 50 % paw withdrawal threshold [[38], [39], [40]].

### Enzyme linked immunosorbent assay (ELISA)

The plasma, DRG, spinal cord, and hippocampus were collected after the mice were anesthetized with isoflurane. The plasma was prepared by centrifuging whole blood for 10 min at 2000 × *g* at 4 °C. Tissues were homogenized and sonicated in radioimmunoprecipitation assay (RIPA) buffer supplemented with a protease inhibitor cocktail (PIC) (Roche, Basel, Switzerland) to ensure protein solubilization. The supernatants were centrifuged at 11000 × *g* for 20 min at 4 °C, and the resulting supernatants were transferred to new tubes for further analysis. Protein quantification was performed using a bicinchoninic acid assay kit (Thermo Fisher Scientific, MA, USA) according to the manufacturer’s instructions.

Aβ_1–42_ levels in the plasma and tissue lysates were measured using the Amyloid Beta 42 Human ELISA Kit (KHB3544, Thermo Fisher Scientific, MA, USA) following the manufacturer’s instructions. The assay sensitivity allowed for the detection of Aβ_1–42_ concentrations as low as 1 pg/mL, and the dilution linearity was validated between 1:2 and 1:16 to ensure consistent results across different sample dilutions. The specificity of the ELISA kit was confirmed, with no cross-reactivity observed with related proteins.

### Primary mouse and human DRG neuron cultures

DRGs were acquired from mice (6–9-weeks-old) and incubated with collagenase A (0.2 mg/mL, Roche)/dispase II (2.4 units/mL, Roche) at 37 °C for 90 min. The cells were mechanically dissociated through gentle pipetting in Dulbecco's modified Eagle’s medium (DMEM, Gibco) with 10 % fetal bovine serum (FBS, Gibco) and 1 % penicillin/streptomycin (P/S, Gibco). Dissociated DRGs were plated on poly-l-lysine-coated coverslips and grown in a neurobasal culture medium with 10 % FBS, 2 % B27 supplement (Invitrogen, Carlsbad, CA, USA), and 1 % P/S for 1 day before calcium imaging and patch-clamp recordings were performed.

Human DRGs were obtained from patients at the [BLINDED FOR REVIEW]. The study involving human DRG tissues received ethical approval from the Institutional Review Board of [BLINDED FOR REVIEW] (approval number: 2018-11-063-002) and all procedures were performed in compliance with the relevant laws and institutional guidelines. Informed consent was obtained from all patients. Postmortem DRGs were dissected from donors and delivered in an ice-cold culture medium to the laboratory at [BLINDED FOR REVIEW] within 24 h. Upon delivery, the DRG was rapidly dissected from the nerve roots, minced in Ca^2+^-free Hank's balanced salt solution (Gibco), and digested at 37 °C in a humidified CO_2_ incubator for 180 min with collagenase type II (Worthington, Lakewood, NJ, USA; 390 units/mg; 12 mg/mL final concentration) and dispase II (Roche; 1 unit/mg, 20 mg/mL) in phosphate-buffered saline (PBS) with 200 μM sodium pyruvate and 10 mM 4-(2-hydroxyethyl)-1-piperazineethanesulfonic acid (HEPES). The pH was adjusted to 7.4 with NaOH. The disintegrated DRGs were centrifuged for 5 min at 400 × *g* to pellet the ganglia. The collagenase II/dispase II solution was carefully removed and the human DRG cells were mechanically dissociated in a DMEM culture medium with 10 % FBS and 1 % P/S. The cell suspension was filtered through a 100 μm nylon mesh and centrifuged for 5 min at 400 × *g*. The DRG cell pellet was resuspended and plated on 0.1 mg/mL Corning® Cell-Tak-coated glass coverslips. The DRG cultures were grown in a neurobasal medium supplemented with 10 % FBS, 2 % B-27 supplement, 1 % N2 supplement, and 1 % P/S for at least 5 days. Human primary DRG neurons were used for up to 4 weeks. The human DRG neurons used in this study are listed in Table 1.

**Table 1:** Human dorsal root ganglion donor data.

**Sex**	**Age (years)**	**Tissue level**
Male	81	C8
Male	55	T10
Female	46	T9
Male	65	C8
Female	60	T5
Female	40	C8

### Cell line culture and transfection

HEK293T cells were cultured in DMEM supplemented with 10 % FBS and 1 % P/S. Transfection was performed with the Lipofectamine 2000 reagent (Invitrogen) 1 day after the subculture. The myc-mouse mini LRP1-green fluorescent protein (GFP) plasmid was provided by C. U. Pietrzik from Johannes Gutenberg University of Mainz. Human TRPV1 was purchased from Origene (Rockville, MD, USA), and human SHP2-EGFP was purchased from Addgene (Watertown, MA, USA).

### Electrophysiology

Whole-cell patch-clamp recordings were performed at 25 °C using MPC-200 manipulators (Sutter Instrument, Novato, CA, USA) and a multiclamp 700B amplifier (Molecular Devices, San Jose, CA, USA). The patch pipettes were pulled from borosilicate capillaries (Chase Scientific Glass, Inc., Rockwood, TN, USA). The resistance of the pipette was 2–5 MΩ, and the series resistance was compensated (>80 %). Data were low-pass-filtered at 2 kHz and sampled at 10 kHz. Voltage clamp recordings were performed at a holding potential of −70 mV. The pipette solution for voltage-clamp experiments contained 122.5 mM K-gluconate, 12.5 mM KCl, 0.2 mM ethylene glycol tetraacetic acid (EGTA), 8 mM NaCl, 2 mM MgATP, 0.3 mM Na_3_GTP, and 10 mM HEPES; the pH was adjusted to 7.3 with KOH. The bath solution contained 140 mM NaCl, 5 mM KCl, 1 mM MgCl_2_, 10 mM HEPES, 10 mM glucose, and 2 mM EGTA. In the clamp mode, the step protocol, involving an increase from 0 pA to 200 pA in increments of 10 pA with a pulse duration of 500 ms, was applied to determine the rheobase.

### Calcium imaging

Cells on poly-l-lysine-coated coverslips were loaded with 2 μM Fura-2 AM (Thermo Fisher Scientific) for 40 min at 37 °C in DMEM, and the coverslip was placed on the recording chamber. They were then mounted on an inverted microscope (Olympus, BX51WI, Tokyo, Japan) and perfused continuously with a balanced bath solution containing 140 mM NaCl, 5 mM KCl, 1 mM CaCl_2_, 2 mM MgCl_2_, 10 mM HEPES, and 10 mM glucose. The solution was adjusted to a pH of 7.4 with NaOH. Calcium imaging was performed at 25 °C. The cells were illuminated using a 175 W xenon arc lamp, and excitation wavelengths (340/380 nm) were selected using a Lambda DG-4 monochromator wavelength changer (Shutter Instrument, Novato, CA, USA). The 340/480 nm fluorescence ratio was measured using digital video microfluorometry with an intensified camera (optiMOS, QImaging, Surrey, BC, USA) coupled with a microscope and software (Slidebook 6, 3i, Intelligent Imaging Innovations, Denver, CO, USA). The perfusion system was driven by gravity and a 1 mL/min flow rate.

### Single-cell reverse transcription (RT)-PCR

The entire single-cell solution was aspirated into a pipette under visual control via negative pressure. DRG neurons were visualized using a 20 × water-immersion objective lens using a microscope (Olympus, BX51WI, Tokyo, Japan), and diameters were measured using the Slidebook 6 software. The pipettes used for the entire neuron harvest had a tip diameter range of approximately 20–35 μm and were filled with RNase OUT (Invitrogen) and UltraPure DNase/RNase-Free Distilled Water. The tip of the pipette was broken, and its contents were released into a reaction tube containing RT reagents. RT was conducted for 60 min at 50 °C (Superscript III, Invitrogen). The synthesized cDNA was used in separate PCRs. All PCR amplifications were performed using nested primers. First-round PCR was performed using cDNA and outer primers to extract each neuron. Subsequently, second-round PCR was performed with the first-round PCR products and inner primers. The sequence of primers used in this study is listed in Table 2.

**Table 2:** Polymerase chain reaction primer sequences.

**Primer name**	**Sequence**	**Size (bp)**
*gapdh* outer F	5′-AGCCTCGTCCCGTAGACAAAA-3′	367
*gapdh* outer R	5′-TTTTGGCTCCACCCCTTCA-3′	367
*gapdh* inner F	5′-TGAAGGTCGGTGTGAACGAATT-3′	313
*gapdh* inner R	5′-GCTTTCTCCATGGTGGTGAAGA-3′	313
*lrp1* outer F	5′-CTGTAGCAATGGTGGCTCCT-3′	453
*lrp1* outer R	5′-AGCAGTTCTCGCTTCTCGTC-3′	453
*lrp1* inner F	5′-TTCTGGTATAAGCGGCGAGT-3′	209
*lrp1* inner R	5′-TAGAGCGTGGCATACACTGG-3′	209
*trpv1* outer F	5′-TGATCATCTTCACCACGGCTG-3′	273
*trpv1 outer R*	5′-CCTTGCGATGGCTGAAGTACA-3′	273
*trpv1 inner F*	5′-AAGGCTTGCCCCCCTATAA-3′	203
*trpv1 inner R*	5′-CACCAGCATGAACAGTGACTGT-3′	203

*gapdh,* glyceraldehyde-3-phosphate dehydrogenase; *lrp1*, low-density lipoprotein receptor-related protein 1; *trpv1*, transient receptor potential vanilloid 1; F; forward; R, reverse.

### Immunohistochemistry

Mouse DRGs were collected after anesthetizing the mice with isoflurane and perfusing them with PBS, followed by 4 % paraformaldehyde (PFA) in PBS. Human DRG were obtained from patients at the [BLINDED FOR REVIEW] (IRB number: 2018-11-063-002) and fixed using 4 % PFA in PBS. The mouse spinal cord tissues were fixed with 4 % PFA for 2 h after extraction. Following dehydration in 30 % sucrose solution overnight at 4 °C, the fixed DRG and spinal cord tissues were sectioned into slices of 14 and 30 μm thicknesses using a cryostat, respectively. The sections were blocked with 5 % FBS for 1 h at 25 °C and incubated overnight at 4 °C with the following primary antibodies: rabbit anti-LRP1 (1:100, Invitrogen, MA5-31959), mouse anti-NeuN (1:100, Millipore, MAB377), mouse anti-NF200 (1:100, Sigma, N0153), mouse anti-calcitonin gene-related peptide (CGRP; 1:100, Abcam, ab81887), mouse anti-isolectin B4 (IB4)-488 (1:100, Invitrogen, 121411), and mouse anti-TRPV1 (1:100, Santacruz, sc-398417) followed by 594- or 488-conjugated secondary antibodies (1:200, Santacruz). The fluorescence of the sections was examined under an LSM700 confocal microscope.

Quantification of immunohistochemistry results was performed using ImageJ (Fiji, Bethesda, MD, USA) with standardized imaging parameters to ensure consistency across samples. Fluorescence intensity was quantified by converting images to 8-bit grayscale at a resolution of 1024 × 1024 pixels to maintain consistency in analysis. Background fluorescence was subtracted using the rolling ball algorithm (∼100 μm) to minimize signal noise. A fixed intensity threshold was applied to segment Regions of Interest (ROIs), and intensity was quantified within these regions. Mean fluorescence intensity was measured per cell using the “Measure” function in ImageJ, and values were normalized to background intensity from adjacent non-stained regions. Colocalization analysis was conducted using the Coloc 2 plugin in ImageJ (Fiji) to determine Pearson’s correlation coefficient for assessing marker colocalization. To validate the consistency and reproducibility, three independent biological replicates (n = 3) with technical replicates were analyzed, and statistical significance was determined using one-way ANOVA.

### RNAscope (fluorescence in situ hybridization)

The mice were anesthetized using isoflurane, and L3–5 DRG tissues were extracted from the spinal column. L3–5 DRG tissues were fixed with 4 % PFA for 2 h and dehydrated in 30 % sucrose overnight at 4 °C. Tissues were sectioned into 12  µm thick slices and mounted on slides. RNA was detected using the RNAscope Multiplex Fluorescent V2 Assay (Advanced Cell Diagnostics, Newark, CA, USA) by omitting the initial on-slide fixation step. The Mm-Lrp1 (Advanced Cell Diagnostics, 465231) and Mm-Trpv1-C2 (Advanced Cell Di415381-C2) probes were used. After probe incubation and fluorescence signal amplification, the slides were incubated in Nissl (Thermo Fisher Scientific, N21483) in PBS for 10 min and mounted using the Prolong Gold Antifade Mounting Medium (Thermo Fisher Scientific, P36930). Images were acquired using a Keyence BZ-X800 microscope and analyzed using the NIH Image J open-source software [41].

### Lentivirus short hairpin RNA (shRNA)-mediated *Lrp1* knockdown

Lentiviral vector plasmids containing the designed shRNA targeting *Lrp1* and negative control genes were obtained by cloning into the pLenti-H1 lentiviral vector expressing the red fluorescent protein. One day after the primary mouse DRG culture, lentiviral shRNA was transduced at 50 multiplicity of infection. After lentiviral infection, infected DRG neurons were observed under a fluorescence microscope (Nickon, Japan) 6 days post-injection.

### Co-immunoprecipitation (Co-IP) and western blotting

Transfected HEK293T cells were lysed in an ice-cold immunoprecipitation buffer (50 mM Tris-HCl, pH 7.4, 100 mM NaCl, 2.134 mM MgCl_2_, 10 mM EDTA, 1 % Triton X-100, and 10 % glycerol) mixed with a PIC. The lysate was precleared with Protein A/G PLUS-Agarose (Santacruz, sc-2003), immunoprecipitated with 10 μg of the mouse anti-GFP antibody (Abcam, ab1218), mouse anti-SHP2 antibody (Santacruz, sc-7384), and normal mouse IgG, and then incubated with Protein A/G PLUS-Agarose. The immunoprecipitates were collected and washed three times with a wash buffer (50 mM Tris-HCl, pH 7.4, 150 mM NaCl, 10 mM EDTA, and 1 % NP-40). For the Co-IP, the blots were incubated with diluted rabbit-pSHP2 (Y542) (1:1000, CST, 3751) and rabbit anti-LRP1 (1:10000, Abcam, ab92544) primary antibodies. The cells were washed with PBS. For the mouse DRG, the mice were anesthetized with isoflurane and perfused with PBS, and DRG tissues were extracted from the spinal column. For human DRG, the tissues were washed with PBS, 200 μM sodium pyruvate, and 10 mM HEPES and finely minced. The cells were sonicated, and tissues were homogenized in a lysis buffer containing PIC. For the phosphorylation, a phosphatase inhibitor was added to the lysis buffer. Quantification was performed using a bicinchoninic acid assay kit (Thermo Fisher Scientific). A phosphatase inhibitor cocktail was added to the lysis buffer to compare phosphorylation. The protein was transferred to a nitrocellulose membrane and blocked with 5 % skim milk or 5 % bovine serum albumin. The blots were incubated with the following primary antibodies: diluted rabbit anti-LRP1 (1:10000, Abcam, ab92544), mouse anti-TRPV1 (1:1000, Santacruz, sc-398417), mouse anti-β-actin (1:1000, Sigma, A2228), rabbit anti-pSHP2 (Y542) (1:1000, CST, 3751), mouse anti-SHP2 (1:1000, Santacruz, sc-7384), and rabbit anti-GAPDH (1:1000, Santacruz, sc-25778). After adding 0.1 % Tween-20 in Tris-buffered saline, the blots were incubated further with a diluted horseradish peroxidase-conjugated secondary antibody (1:2000, Santacruz) and developed in an enhanced luminol-based chemiluminescent substrate solution. Specific bands were evaluated based on their apparent molecular sizes.

### Statistical analyses

All data are expressed as mean ± standard error of the mean. In vivo experimental, biochemical, electrophysiological, and calcium imaging data were analyzed using the Student's *t*-test (two groups) and one- or two-way analysis of variance followed by the Sidak post-hoc test using Prism 8 software (GraphPad Software, San Diego, CA, USA). A *p*-value of < 0.05 was considered statistically significant.

## Results

### Elevated Aβ_1–42_ levels induce a reduction in heat pain sensitivity in mature adult mice

**Fig. 1:**
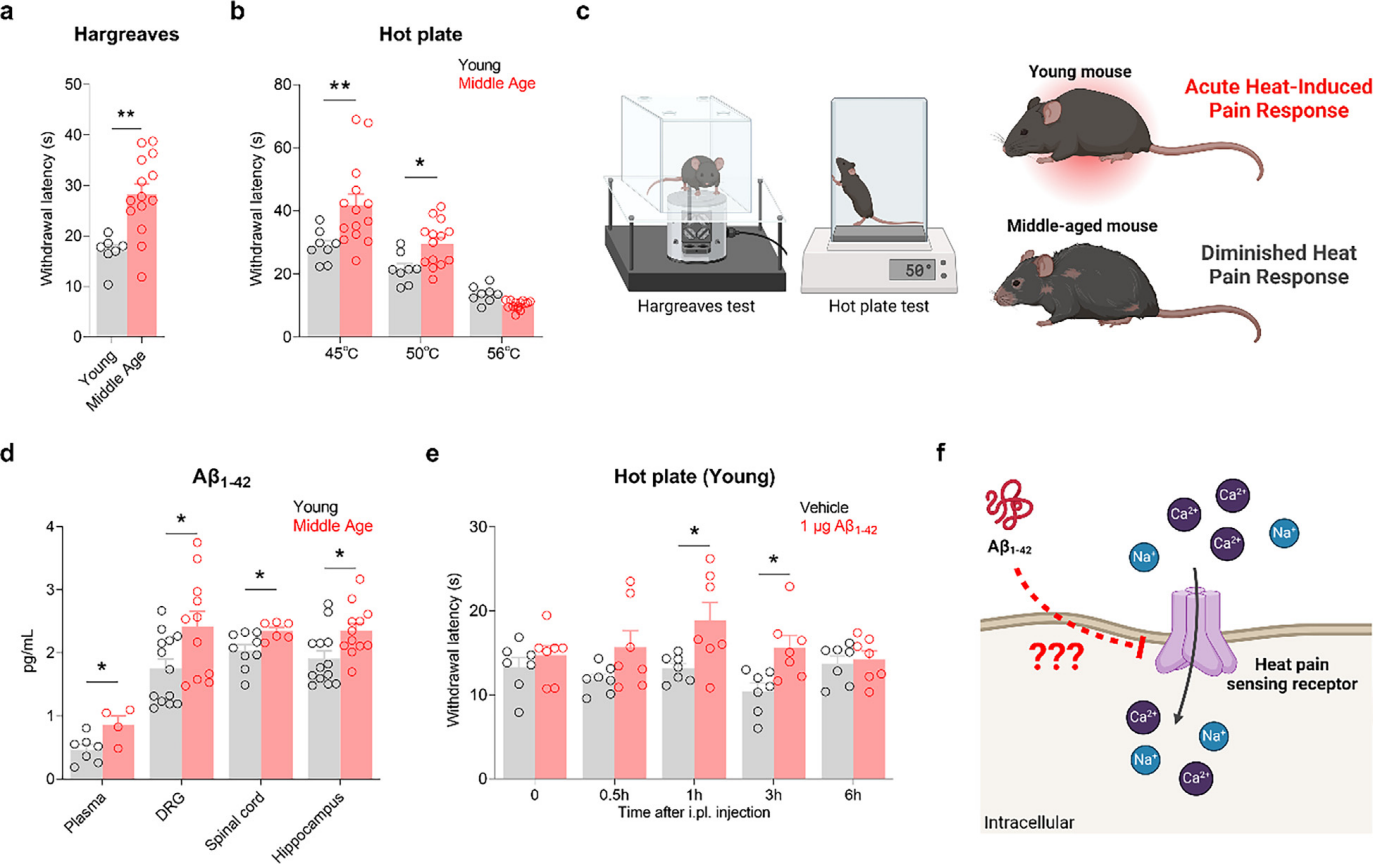
**Mature adult mice exhibit reduced heat pain sensitivity associated with increased Aβ_1–42_ levels; Aβ_1–42_ treatment reduces heat pain sensitivity in naïve young mice**. **a, b:** The Hargreaves test (a) and hot plate test (b) were used to assess heat pain sensitivity at baseline in naïve young and mature adult mice. *p < 0.05, **p < 0.01, unpaired *t*-test; young, n = 7–8; mature adults, n = 14. **c:** Schematic representation of the Hargreaves and hot plate tests used to assess heat pain sensitivity in young and mature adult mice. **d:** Enzyme linked immunosorbent assay showing endogenous expression of Aβ_1–42_ in the plasma, dorsal root ganglion, spinal cord, and hippocampus of young and mature adult mice. *p < 0.05, unpaired *t*-test; young, n = 7–13; mature adults, n = 4–12. **e:** Hot plate test to examine basal heat sensitivity after intraplantar injection of 1 μg Aβ_1–42_ in young mice. *p < 0.05, two-way analysis of variance with the Sidak post-hoc test, n = 7/group. **f:** Proposed model illustrating how Aβ_1–42_ may interact with heat pain sensing receptors to reduce heat pain sensitivity. Data are expressed as mean ± standard error of the mean.

To explore the impact of Aβ_1–42_ on heat pain sensitivity, we performed thermosensory assessments, including the Hargreaves and hot plate tests, on young and mature adult mice (Fig. 1). The behavioral data revealed a notable disparity. Mature adult mice exhibited significantly higher withdrawal latencies and delayed responses to noxious heat, such as paw licking, lifting, shaking, or jumping (Fig. 1a and b). Collectively, these findings demonstrate a clear difference in thermal nociception, with mature adult mice showing reduced heat-induced pain responses.

To further investigate the potential role of Aβ_1–42_ in modulating pain sensitivity, we measured its concentration in various tissues, including the DRG, plasma, spinal cord, and hippocampus, using ELISA. The Aβ_1–42_ level was found to be significantly elevated in all tissues in mature adult mice compared with that in their younger counterparts (Fig. 1d). The level of Aβ_1–42_ in mature adults was correlated with the heat pain threshold. Moreover, we investigated the effect of Aβ_1–42_ on heat pain sensitivity in young mice by administering intraplantar injections, following which, the mice displayed a reduction in heat pain sensitivity, mirroring the responses observed in mature adult mice (Fig. 1e). However, additional experiments revealed that Aβ_1–42_ treatment in young female mice had no significant effect on heat pain sensitivity (Supplementary Fig. 1), suggesting a potential sex-dependent response to Aβ_1–42_. This result suggests that elevated Aβ_1–42_ levels are associated with reduced heat pain sensitivity in mature adult mice and can modulate sensitivity in younger mice in similar manner by interacting with heat pain sensing receptors (Fig. 1f).

### Aβ_1–42_ inhibits TRPV1 activation via LRP1 in mice DRG neurons

**Fig. 2:**
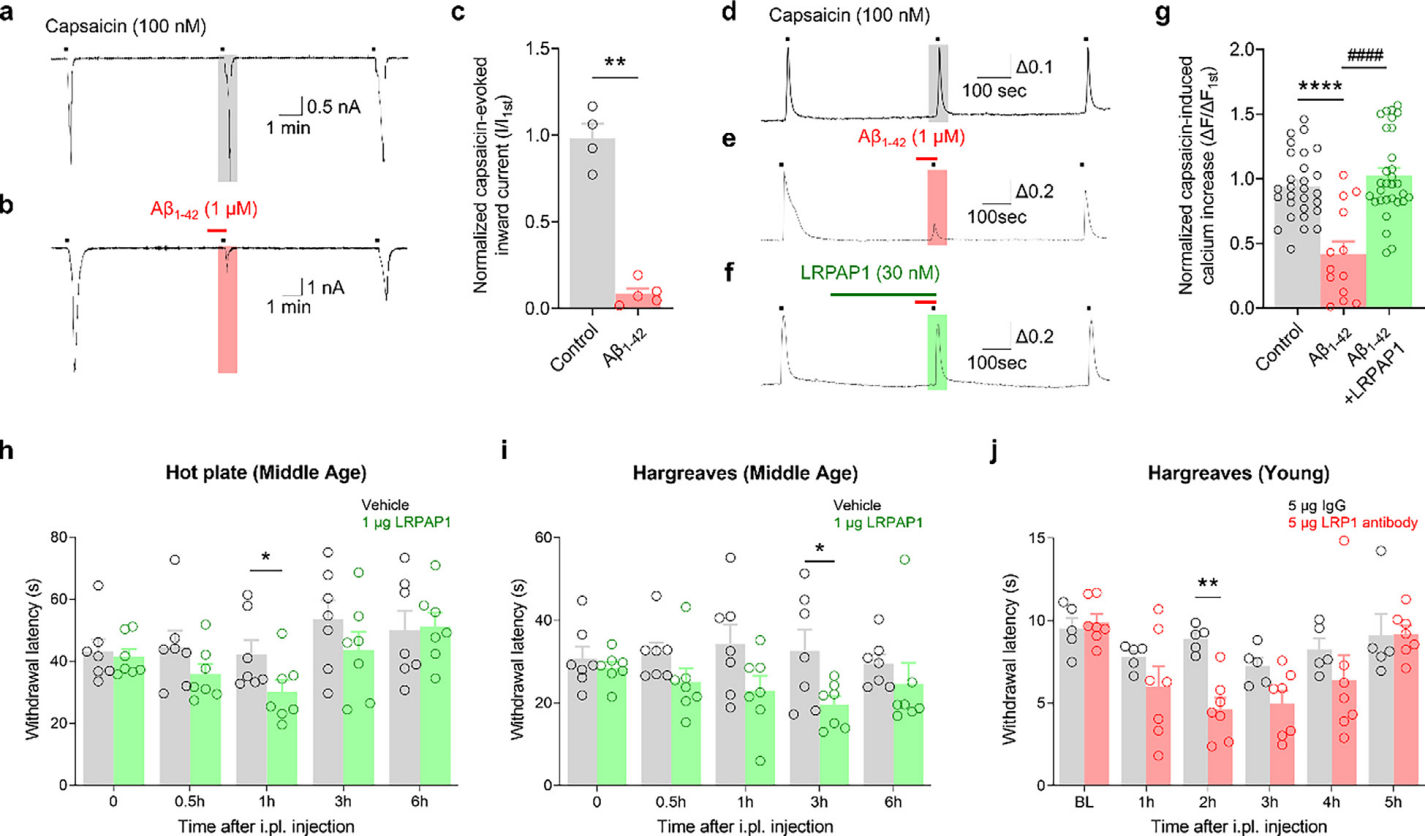
**Aβ_1–42_ suppresses TRPV1 activation in mouse DRG neurons and in a capsaicin-induced acute pain model via LRP1: Electrophysiological and calcium imaging studies. a**–**c:** Representative traces (a, b) and bar graph (c) showing 100 nM capsaicin-evoked inward currents after 1 μM Aβ_1–42_ injection in mouse DRG (mDRG) neurons. **p < 0.01, unpaired *t*-test; control, n = 4; Aβ_1–42_, n = 5. **d**–**g:** Representative traces (d–f) and bar graph (g) show a 100 nM capsaicin-induced Ca^2+^ increase after 1 μM Aβ_1–42_ injection following 30 nM LRPAP1 pretreatment in mDRG neurons. ****^, ####^*p* < 0.0001, one-way ANOVA; control, n = 28; Aβ_1–42_, n = 13; Aβ_1–42_ + LRPAP1, n = 30. **h, i:** Hot plate and Hargreaves tests for basal heat sensitivity after intraplantar injection of 1 μg LRPAP1 in mature adult mice. *p < 0.05, two-way ANOVA with the Sidak post-hoc test, n = 7/group. **g:** Hargreaves test for basal heat sensitivity after intraplantar injection of 5 μg LRP1-neutralizing antibody in young mice. **p < 0.01, two-way ANOVA with Sidak post-hoc test; IgG, n = 5; LRP1 antibody, n = 7. Data are expressed as mean ± SEM. DRG, dorsal root ganglion; LRP1, low-density lipoprotein receptor-related protein 1; TRPV1, transient receptor potential vanilloid 1; LRPAP1, low-density lipoprotein receptor-related protein-associated protein 1; ANOVA, analysis of variance; SEM, standard error of the mean.

After discovering that Aβ_1–42_ alters heat pain sensitivity, we sought to determine whether these modulatory effects originate in peripheral sensory neurons. Specifically, we focused on the role of TRPV1 in DRG neurons. We used electrophysiological and calcium imaging techniques to observe the neuronal responses to capsaicin, a selective TRPV1 agonist. An notable decrease in capsaicin-triggered currents and calcium influx caused by Aβ_1–42_ was detected (Fig. 2a–g), indicating distinct modulation of TRPV1. Interestingly, Aβ_1–42_ did not alter the frequency of action potentials, resting membrane potentials, or rheobases, pointing to its specific action on TRPV1 (Supplementary Fig. 2a–c).

In contrast, pretreatment of DRG neurons with the LRP1 antagonist LRPAP1 completely negated the inhibitory effect of Aβ_1–42_ on capsaicin-induced increases in calcium (Fig. 2d–g), confirming the crucial role of LRP1 in Aβ_1–42_-mediated TRPV1 regulation. We then examined the function of LRP1 in pain sensitivity in mature adult mice in vivo. Intraplantar injections of LRPAP1 significantly decreased the heat pain withdrawal latency in the hot plate and Hargreaves tests (Fig. 2h–i), suggesting a direct association between LRP1 activity and heat pain perception. However, mechanical allodynia remained unaffected by LRPAP1 (Supplementary Fig. 3a). Contrarily, intrathecal injections of Aβ_1–42_ alleviated both heat hyperalgesia and mechanical allodynia; these effects were reversed when Aβ_1–42_ was co-administered with LRPAP1 (Supplementary Fig. 3b and c). Finally, we examined the regulatory role of LRP1 by administering a neutralizing antibody against LRP1 in young mice and found increased heat sensitivity (Fig. 2j). These findings collectively establish that Aβ_1–42_ modulates TRPV1 activation through LRP1 signaling, contributing to the maturation-associated variations in heat pain sensitivity.

### LRP1 is co-expressed with TRPV1 in mouse DRG neurons

**Fig. 3:**
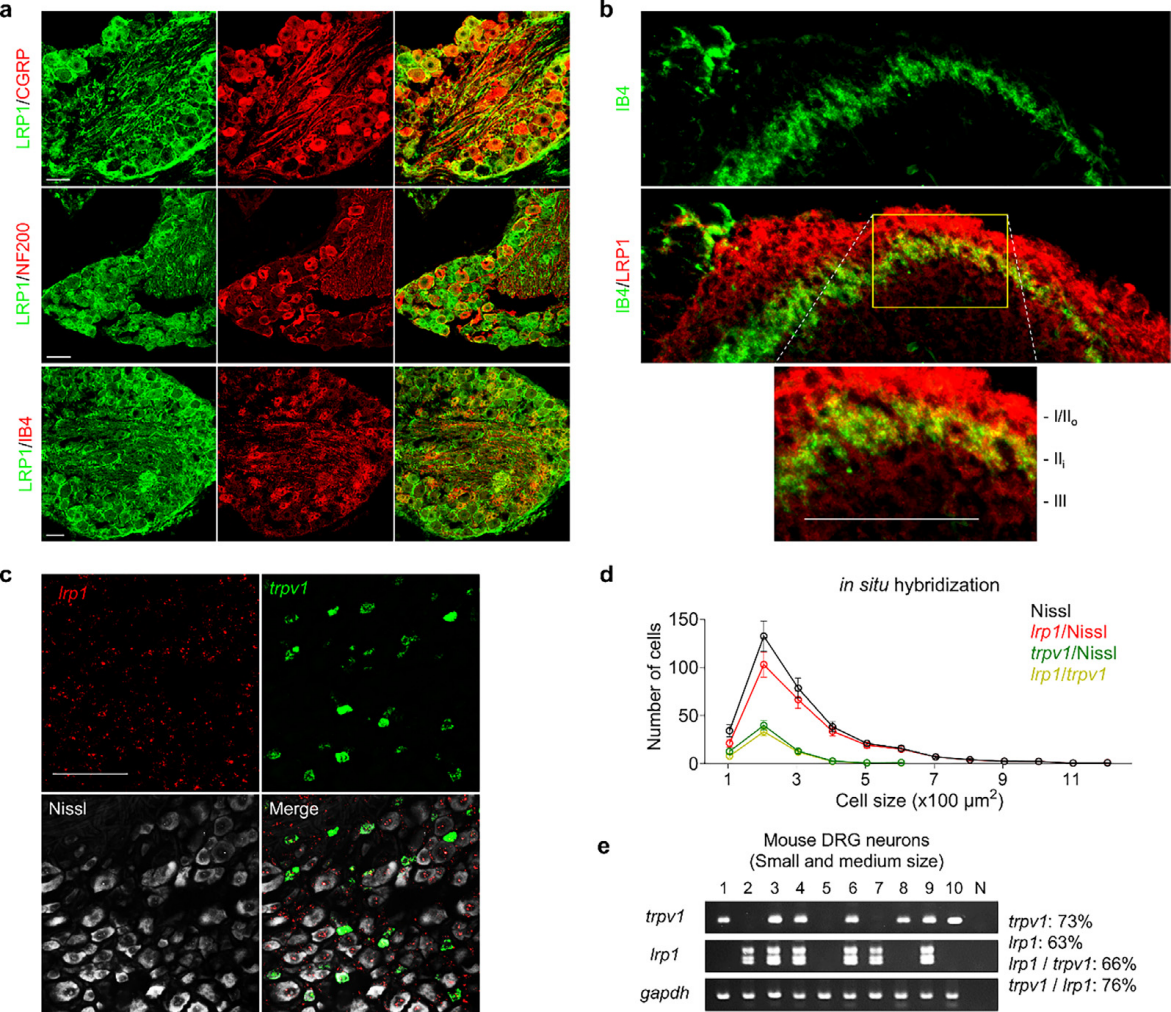
**LRP1 is co-expressed with TRPV1 in mouse DRG neurons**: **Immunohistochemical analysis**. **a:** Immunohistochemistry showing LRP1 expression in DRG slices, n = 3 (NeuN: neuronal marker, NF200: large-sized-neuronal marker, CGRP: peptidergic small-sized neuronal marker, IB4: nonpeptidergic small-sized neuronal marker). Scale bar: 50 μm. **b:** Immunohistochemistry showing LRP1 and IB4 on L4-spinal cord sections, n = 3. Scale bar, 100 μm. **c:** In situ hybridization showing *Lrp1* and *Trpv1* expression in DRG slices, n = 3. Scale bar: 100 μm. **d:** Size frequency distribution of *Lrp1* and *Trpv1*-positive and total neurons in mouse DRGs. **e:** Single-cell RT-PCR showing *Lrp1* and *Trpv1*gene co-expression in mDRG neurons. Data are expressed as mean ± SEM. DRG, dorsal root ganglion; LRP1, low-density lipoprotein receptor-related protein 1; TRPV1, transient receptor potential vanilloid 1; CGRP, calcitonin gene-related peptide; IB4, isolectin B4; SEM, standard error of the mean.

We further explored the co-expression patterns of LRP1 and TRPV1 within mouse DRG neurons by immunohistochemical analysis (Fig. 3). Significant LRP1 expression was detected in various subpopulations of DRG neurons. Notably, double staining techniques revealed a pronounced presence of LRP1 in both small- and medium-sized peptidergic (CGRP+) and non-peptidergic (IB4+) neurons, as well as in large myelinated neurons (NF200+) (Fig. 3a). Further observations highlighted LRP1 localization within the superficial layers of the spinal cord (laminae I–II), which are known to harbor interneurons that connect with nociceptive DRG sensory neurons (Fig. 3b).

We used in situ hybridization and gene expression analyses to confirm the widespread expression of LRP1 in DRG neurons of varying sizes, with notable co-localization observed in most DRG neurons expressing TRPV1 (Fig. 3c–e). These findings collectively underscore the co-expression of LRP1 and TRPV1 in small- and medium-sized mouse DRG neurons.

### LRP1 mediates TRPV1 inhibition by Aβ_1–42_ in mouse DRG neurons

**Fig. 4:**
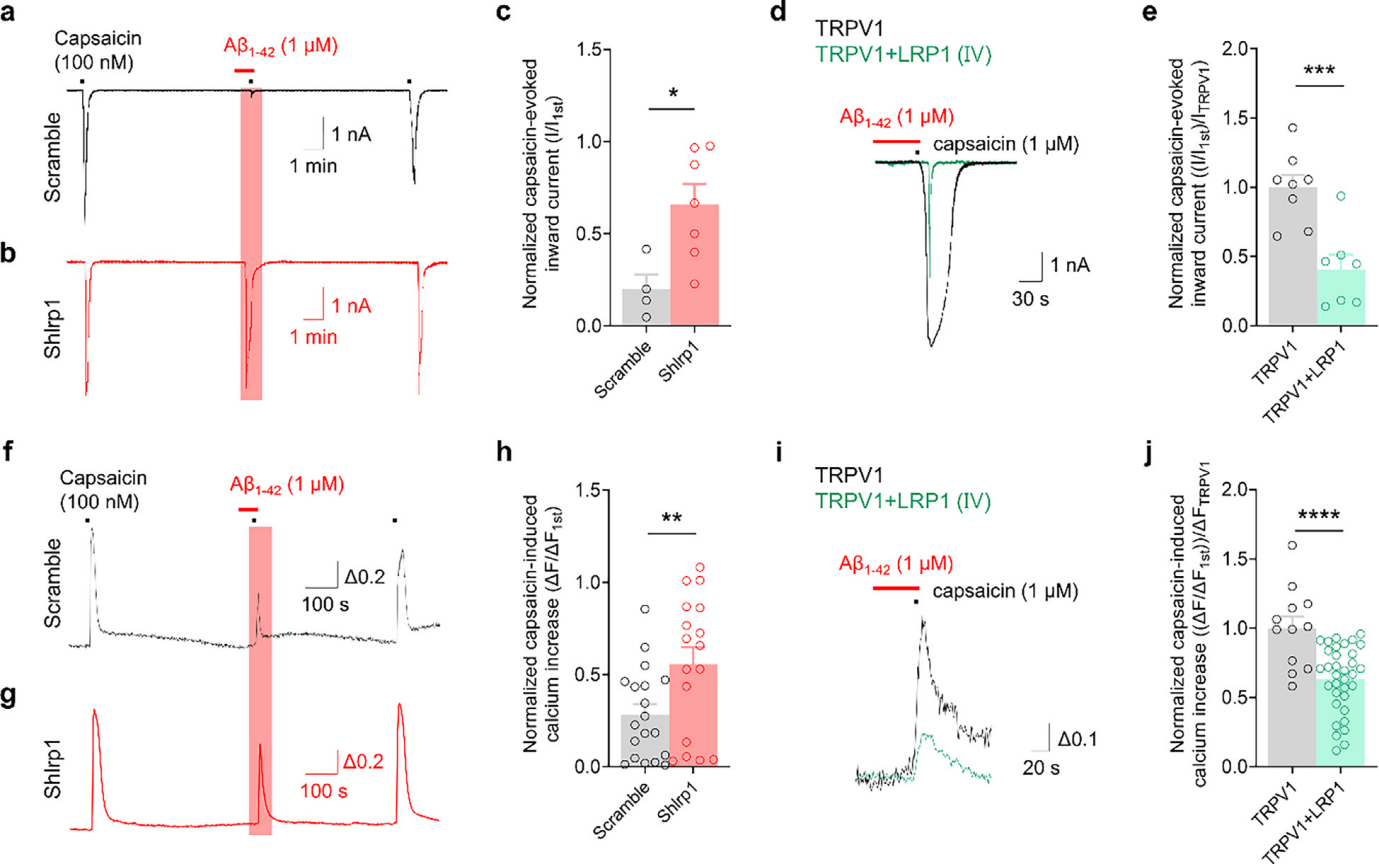
**Knockdown of LRP1 abolishes inhibition of TRPV1 activation by Aβ_1–42_ in mouse DRG neurons. a**–**c:** Representative traces (a, b) and bar graph (c) showing 100 nM capsaicin-evoked inward currents after 1 μM Aβ_1–42_ treatment in mouse DRG (mDRG) neurons. **p < 0.01, unpaired *t*-test; control, n = 4; Aβ_1–42_, n = 5. **d:** Representative traces showing 1 μM capsaicin-evoked inward currents after 1 μM Aβ_1–42_ treatment in TRPV1 (black)- and TRPV1 + LRP1 (turquoise)-expressing HEK293T cells. **e:** Bar graph showing the inward currents normalized by the first inward current. ***p < 0.001, unpaired *t*-test; TRPV1, n = 8; TRPV1/LRP1, n = 7. **f**–**h:** Representative traces (f, g) and a bar graph (h) show the 100 nM capsaicin-induced Ca^2+^ increase after 1 μM Aβ_1–42_ treatment following 30 nM LRPAP1 pretreatment in mDRG neurons. ****^, ####^*p* < 0.0001, one-way ANOVA; control, n = 28; Aβ_1–42_, n = 13; Aβ_1–42_ + LRPAP1, n = 30. **i:** Representative traces showing the 1 μM capsaicin-induced Ca^2+^ increase after 1 μM Aβ_1–42_ treatment in TRPV1 (black)- and TRPV1 + LRP1 (turquoise)-expressing HEK293T cells. **j:** Bar graph showing the Ca^2+^ increases normalized by the first Ca^2+^ increase. ****p < 0.0001, unpaired *t*-test; TRPV1, n = 12; TRPV1/LRP1, n = 34. Data are expressed as mean ± SEM. DRG, dorsal root ganglion; LRP1, low-density lipoprotein receptor-related protein 1; TRPV1, transient receptor potential vanilloid 1; LRPAP1, low-density lipoprotein receptor-related protein-associated protein 1; ANOVA, analysis of variance; SEM, standard error of the mean.

To establish the functional role of LRP1 in moderating TRPV1 activation via Aβ_1–42_, we conducted gene knockdown experiments in DRG cultures. Using shRNA lentiviruses, we aimed to determine the influence of LRP1 in the Aβ_1–42_-mediated inhibition of TRPV1 (Supplementary Fig. 4a and b). In DRG neurons transduced with scrambled shRNA, we found that Aβ_1–42_ pretreatment led to a substantial decrease in capsaicin-induced inward currents. However, this decrease was not observed in neurons subjected to shLRP1 knockdown (Fig. 4a and b). The amplitude of inward currents was significantly higher in the shLRP1 knockdown group than in the scrambled group (Fig. 4c). Further calcium imaging experiments corroborated these results. We noted a significantly higher capsaicin-induced calcium increase in the shLRP1 knockdown group than in the scrambled group (Fig. 4f–h), indicating the crucial role of LRP1 in mediating the inhibitory effect of Aβ_1–42_ on TRPV1. We also examined the difference in capsaicin-induced inward current and calcium increases in HEK293 cells expressing TRPV1 alone versus those co-expressing TRPV1 and LRP1 following Aβ_1–42_ pretreatment. Mirroring the pattern observed in mouse DRG neurons (Fig. 2a–g), we observed that Aβ_1–42_ pretreatment resulted in a reduction of TRPV1 activity in HEK293T cells expressing both TRPV1 and LRP1 but not in those expressing TRPV1 alone (data not shown). Compared with that in HEK293T cells expressing only TRPV1, the secondary TRPV1 activity was significantly diminished in cells co-expressing LRP1 and TRPV1 (Fig. 4d–e and i–j). These experiments clearly indicate that LRP1 is involved in the reduction of TRPV1 activity by interacting with Aβ_1–42_, highlighting its crucial role in the complex mechanisms of nociception.

### Aβ_1–42_ inhibits TRPV1 activation through SHP2 phosphorylation

To explore the complex interaction between Aβ_1–42_ and TRPV1, we investigated the role of SHP, particularly SHP2, in this process. Specifically, we examined the potential of SHP inhibitors to modulate the Aβ_1–42_-induced inhibition of capsaicin-evoked calcium level elevations in neurons.

**Fig. 5:**
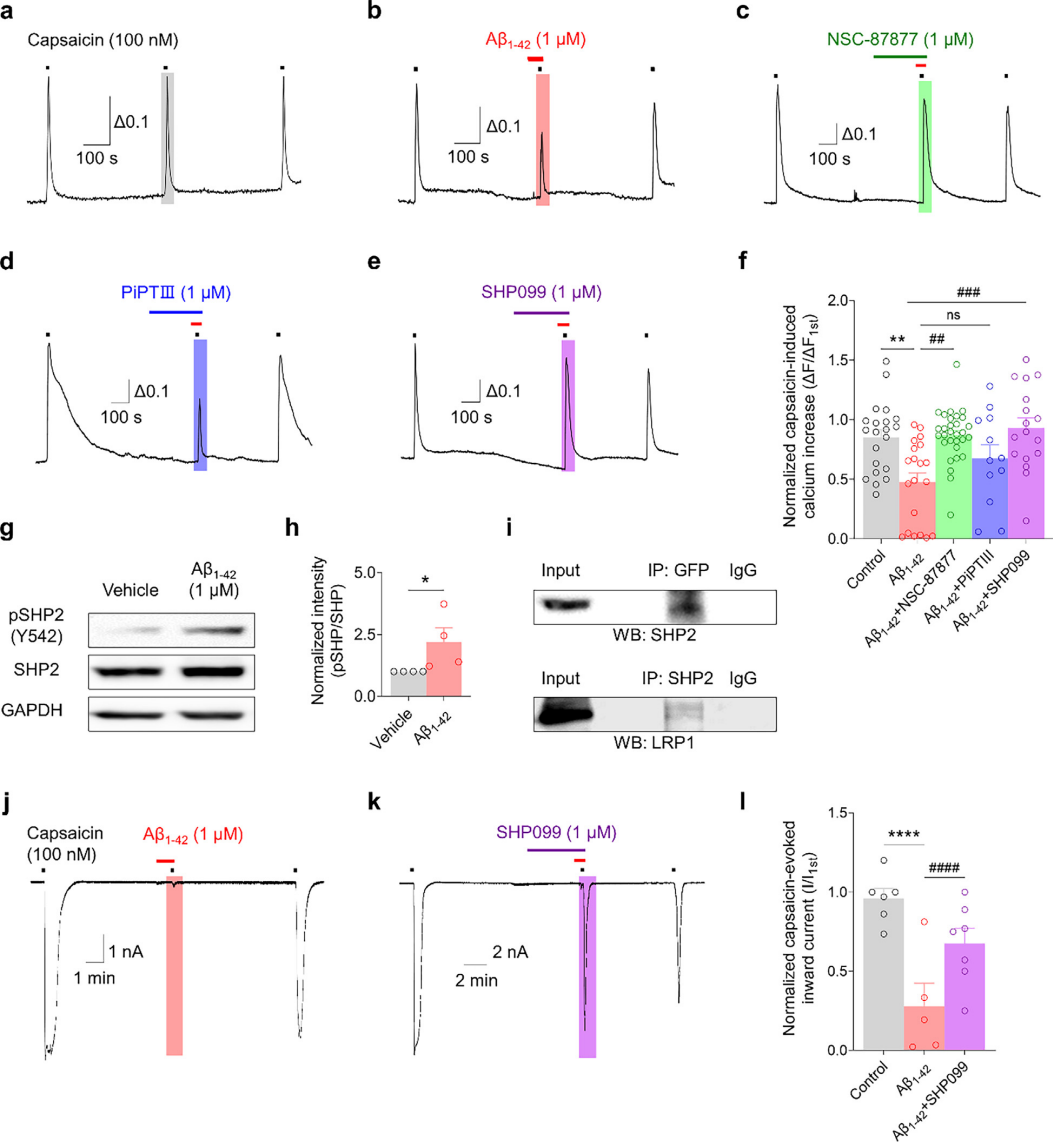
**Aβ_1–42_ modulates TRPV1 activation via SHP2: calcium imaging, western blot, and electrophysiology studies. a–f:** Representative traces (a–e) and a bar graph (f) showing the 100 nM capsaicin-induced Ca^2+^ increase after 1 μM Aβ_1–42_ treatment following 1 µM SHP inhibitor (NSC-87877: pan-SHP inhibitor [green], PiPTⅢ: SHP1 inhibitor [blue], SHP099: SHP2 inhibitor [purple]) pretreatment in mouse DRG (mDRG) neurons. **p < 0.01, ^##^*p* < 0.01, ^###^*p* < 0.001, one-way ANOVA; control, n = 21; Aβ_1–42_, n = 20; Aβ_1–42_ + NSC87877, n = 30; Aβ_1–42_ + PTPiIII, n = 12; Aβ_1–42_ + SHP099, n = 17. **g:** Bands showing an increase of phospho-SHP2 (Y542) levels at 30 min after Aβ_1–42_ (1 μM) treatment in LRP1-expressing HEK293T cells. **h:** Bar graph showing normalized phospho-SHP2 (Y542) intensity. **p < 0.01, unpaired *t*-test, n = 4/group. Data are expressed as mean ± SEM. **i:** Co-immunoprecipitation showing LRP1/SHP2 interaction in TRPV1/SHP2-expressing HEK293T cells. **j**–**l:** Representative traces (j–k) and a bar graph (l) showing 100 nM capsaicin-evoked inward currents after 1 μM SHP099 pretreatment in mDRG neurons. ****^, ####^*p* < 0.0001, one-way ANOVA; control, n = 6; Aβ_1–42_, n = 5; Aβ_1–42_ + SHP099, n = 7. DRG, dorsal root ganglion; LRP1, low-density lipoprotein receptor-related protein 1; TRPV1, transient receptor potential vanilloid 1; SHP2, src-homology domain-2–containing protein tyrosine phosphatase; ANOVA, analysis of variance; SEM, standard error of the mean. (For interpretation of the references to colour in this figure legend, the reader is referred to the web version of this article.)

In our assays, the pan-SHP inhibitor NSC-87877 effectively reversed the TRPV1 inhibition caused by Aβ_1–42_ (Fig. 5a–c). In order to identify the particular SHP isoform involved, we utilized the SHP1 and SHP2 inhibitors, PTPiIII and SHP099, respectively. Interestingly, SHP099, but not PTPiIII, blocked the inhibitory effect of Aβ_1–42_ on the capsaicin-induced calcium increase (Fig. 5d–f). This pattern of inhibition was further supported by patch-clamp results, where SHP099 negated the Aβ_1–42_-mediated suppression of capsaicin-evoked inward currents (Fig. 5j–k). We then examined the impact of Aβ_1–42_ on SHP2 protein expression. Western blot analysis demonstrated increased phosphorylated SHP2 levels following Aβ_1–42_ treatment without affecting total SHP2 levels (Fig. 5g–h). Co-IP experiments in LRP1-expressing HEK293T cells confirmed a physical interaction between LRP1 and phosphorylated SHP2 (Fig. 5i). These findings clearly illustrate how Aβ_1–42_ modulates TRPV1 via SHP2 phosphorylation. Further, these findings reveal a novel pathway in sensory neuron signaling and potential targets for pain management.

### Aβ_1–42_ modulates TRPV1 activation via the endogenous LRP1 pathway in human DRG neurons

**Fig. 6:**
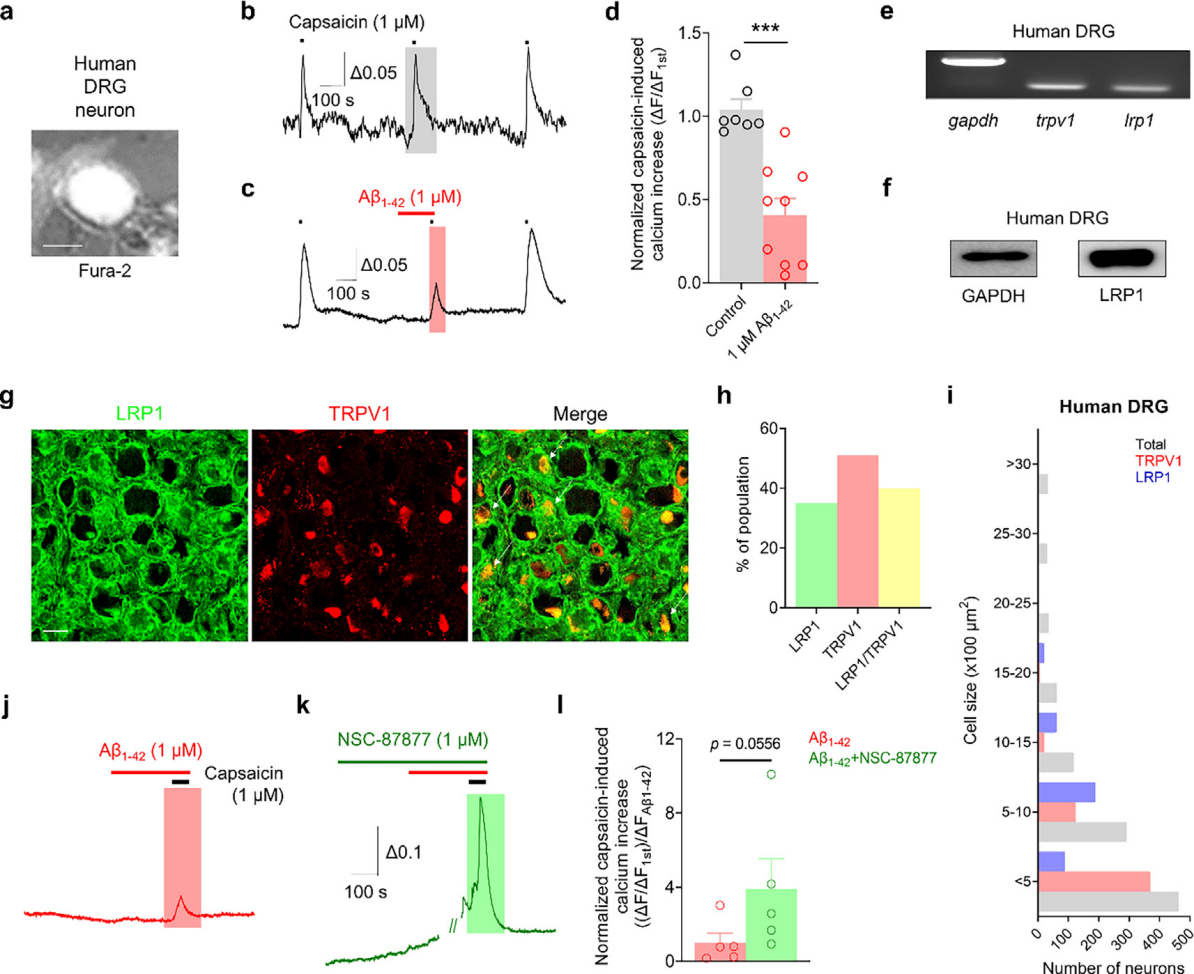
**Aβ_1–42_ modulates TRPV1 activation via SHP2 in human DRG neurons. a:** Human DRG (hDRG) neurons loaded with Fura-2. Scale bar = 25 μm. **b**–**d:** Representative traces (b–c) and a bar graph (d) show a 1 μM capsaicin-induced Ca^2+^ increase after 1 μM Aβ_1–42_ treatment in hDRG neurons. ***p < 0.001, unpaired *t*-test; control, n = 7; Aβ_1–42_, n = 9. **e:** RT-PCR showing *Trpv1* and *Lrp1* gene expression in hDRG tissues. **f:** Western blot showing TRPV1 and LRP1 protein expression in hDRG tissues. **g:** Immunohistochemistry image showing TRPV1 and LRP1 expression in hDRG slices. Scale bar = 100 μm. **h:** Bar graph showing the percentage of the LRP1, TRPV1, and LRP1/TRPV1 population in total hDRG neurons. **i:** Size distribution of LRP1 and TRPV1-positive and total neurons in hDRG. **j**–**l:** Representative traces (j, k) and a bar graph (l) showing the 1 μM capsaicin-induced Ca^2+^ increase after 1 μM Aβ_1–42_ treatment following 1 μM NSC-87877 pretreatment in hDRG neurons. *p* = 0.0556, unpaired *t*-test, n = 5/group. Data are expressed as the mean. DRG, dorsal root ganglion; LRP1, low-density lipoprotein receptor-related protein 1; TRPV1, transient receptor potential vanilloid 1.

We extended our investigation to human DRG neurons to confirm the role of Aβ_1–42_ in modulating TRPV1 activation. Our initial assays demonstrating significant inhibition of capsaicin-induced intracellular calcium elevation in primary human DRG neurons by Aβ_1–42_ (Fig. 6a–d) prompted us to examine the expression of LRP1 in these neurons. Both mRNA and protein expression analyses confirmed the presence of LRP1 in human DRG neurons (Fig. 6e–f). Immunohistochemical (IHC) studies further revealed notable expression of LRP1 (45 %) and TRPV1 (50 %) in small- and medium-sized human DRG neurons, with significant overlap (50 %) between LRP1 and TRPV1 expression (Fig. 6g–i). This co-expression suggests a potentially complex interaction between these two proteins in human sensory neurons. We evaluated the effects of the pan-SHP inhibitor NSC-87877 on the modulation of capsaicin-induced calcium responses by Aβ_1–42_. The results indicated that NSC-87877 could disrupt the inhibitory effect of Aβ_1–42_ on human DRG neurons (Fig. 6j–l), reinforcing the hypothesis that Aβ_1–42_ modulates TRPV1 activation via the endogenous LRP1 pathway in human sensory neurons.

### Verification of TRPV1 inhibition by LRP1 using a potent LRP1 agonist, α_2_ macroglobulin

**Fig. 7:**
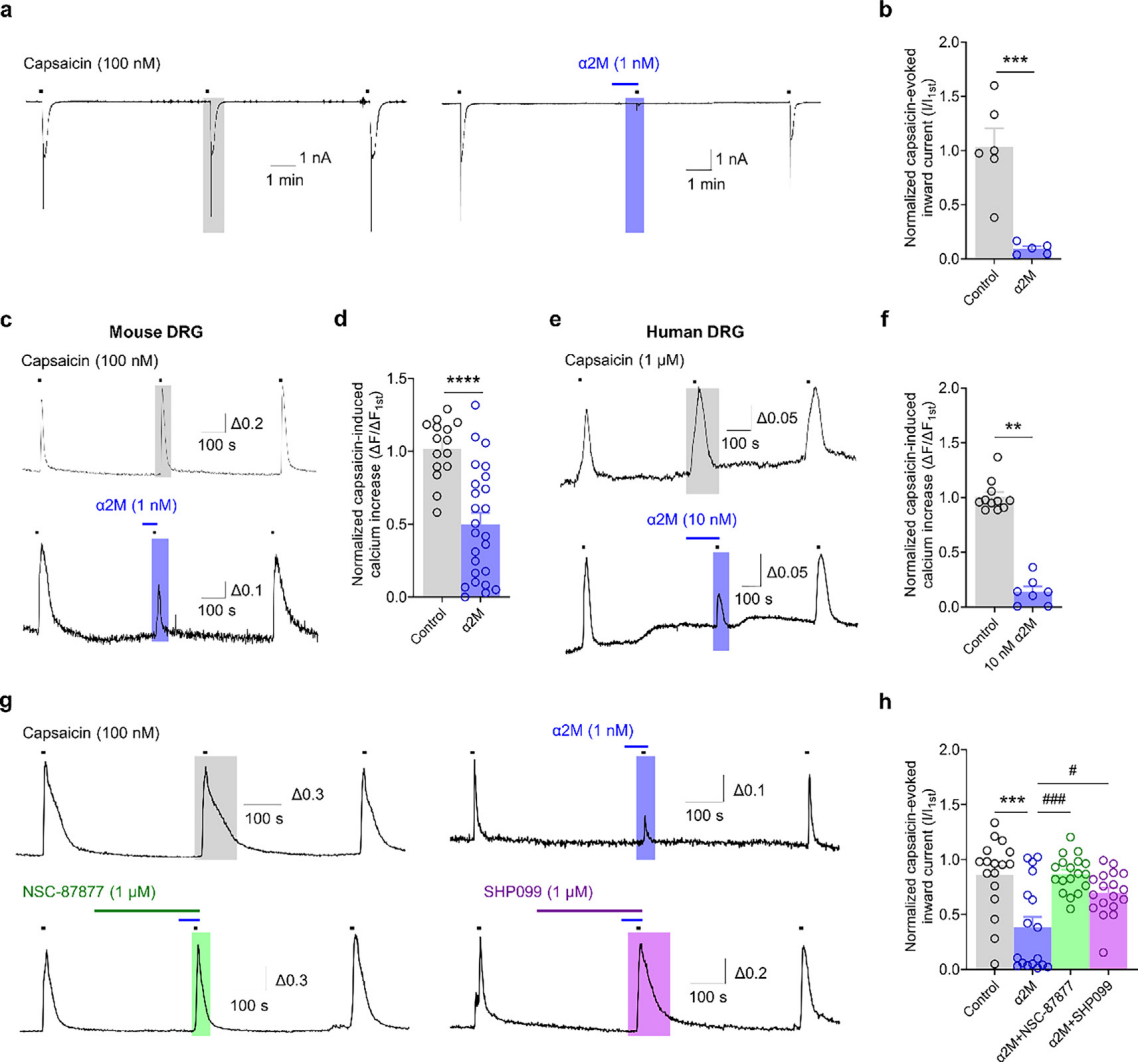
**Aβ_1–42_ modulates TRPV1 activation via SHP2. a–b:** Representative traces (a) and a bar graph (b) showing the 100 nM capsaicin-evoked inward currents after 1 nM α_2_M injection in mouse DRG (mDRG) neurons. ***p < 0.001, unpaired *t*-test; control, n = 4; α2M, n = 5. **c–d:** Representative traces (c) and a bar graph (d) show the 100 nM capsaicin-induced Ca^2+^ increase after 1 nM α_2_M injection in mDRG neurons. ***p < 0.001, one-way ANOVA; control, n = 20; α2M, n = 20. **e–f:** Representative traces (e) and a bar graph (f) show the 1 μM capsaicin-induced Ca^2+^ increase after 1 nM α_2_M treatment in hDRG neurons. ****p < 0.0001, unpaired *t*-test; control, n = 7; α_2_M, n = 7. **g–h:** Representative traces (g) and a bar graph (h) showing the 100 nM capsaicin-induced Ca^2+^ increase after 1 nM α_2_M treatment following 1 µM SHP inhibitor (NSC-87877: pan-SHP inhibitor [green], SHP099: SHP2 inhibitor [purple]) pretreatment in mouse DRG (mDRG) neurons. ^#^*p* < 0.05, ^***^p < 0.001, ^###^*p* < 0.001, one-way ANOVA; control, n = 20; α_2_M, n = 20; α_2_M + NSC87877, n = 20; α_2_M + SHP099, n = 20. DRG, dorsal root ganglion; TRPV1, transient receptor potential vanilloid 1; SHP2, src-homology domain-2–containing protein tyrosine phosphatase; α_2_M, α_2_-macroglobulin; ANOVA, analysis of variance. (For interpretation of the references to colour in this figure legend, the reader is referred to the web version of this article.)

Previous studies have revealed that α_2_M is a potent LRP1 agonist [32]. Therefore, we used α_2_M to examine whether the inhibitory and analgesic effects of Aβ_1-42_ on TRPV1 activity are mediated through LRP1. We found that α_2_M induced a marked decrease in the capsaicin-induced inward current (Fig. 7 a–b). Further, as shown in Fig. 7c–f, α_2_M inhibited the capsaicin-induced intracellular calcium increase in mouse and human DRG neurons. Next, we investigated whether α2M exerts its inhibitory effect on TRPV1 activity through SHP2 signaling. We found that NSC-87877 and SHP099 effectively reversed the inhibitory effect of α2M on the capsaicin-induced calcium increase (Fig. 7 g–h). Moreover, we observed that α_2_M significantly increased SHP2 phosphorylation to a similar extent compared with Aβ_1-42_ (data not shown), indicating that the in vitro LRP1/SHP2 signaling corroborates the experimental findings observed with Aβ_1-42_.

### Aβ_1–42_ modulates heat hyperalgesia in the mouse SNI model via the LRP1-SHP2 pathway

**Fig. 8:**
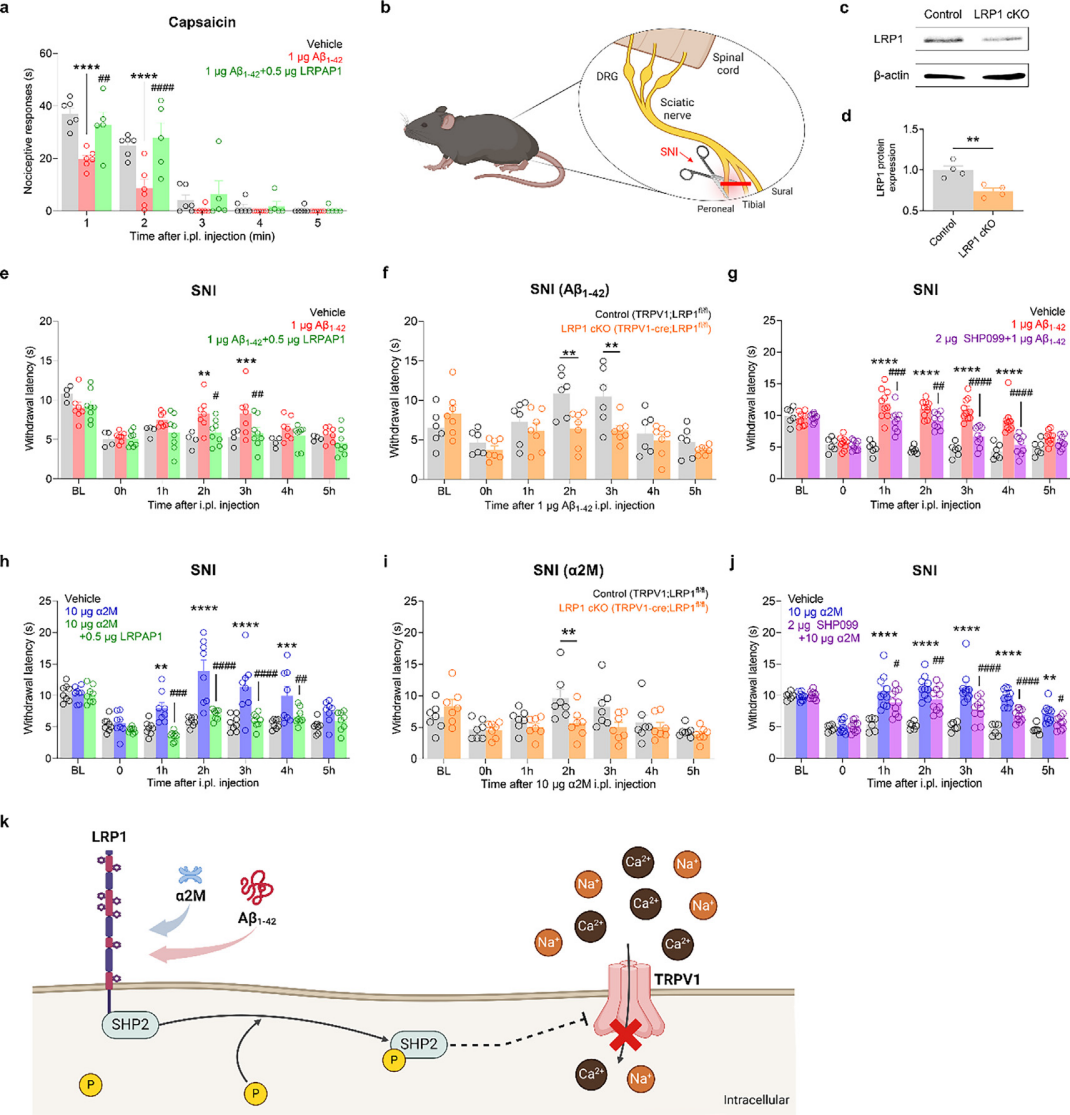
**Aβ_1–42_ and α_2_M modulate heat hyperalgesia in an SNI model via the LRP1-SHP2 pathway. a:** Nociceptive responses evoked by intraplantar injection of 0.125 μg capsaicin with 1 μg Aβ_1–42_ and 0.5 μg LRPAP1. ^#^*p* < 0.05, **^, ##^*p* < 0.01, ****^, ####^*p* < 0.0001, two-way ANOVA with the Sidak post-hoc test. **b:** Schematic illustration of the spared nerve injury (SNI) model used to induce neuropathic pain in mice. **c:** Bands showing LRP1 protein expression after injection of AAV5-control (black) and AAV5-cre (orange) in mouse DRG. **d:** The bar graph shows normalized band intensity (n = 4). **p < 0.01. **e:** Heat hyperalgesia in the SNI chronic pain model following intraplantar injection of 1 μg Aβ_1–42_ and 0.5 μg LRPAP1. ^#^*p* < 0.05, **^,^^##^*p* < 0.01, ***p < 0.001, two-way ANOVA with the Sidak post-hoc test; vehicle, n = 4; Aβ_1–42_, n = 7; Aβ_1–42_ + LRPAP1, n = 8. **f:** SNI-induced heat hyperalgesia in control and LRP1 cKO mice following intraplantar injection of 1 μg Aβ_1–42_. **p < 0.01, two-way ANOVA with the Sidak post-hoc test; control, n = 6; LRP1 cKO, n = 7. **g:** Heat hyperalgesia in the SNI chronic pain model following intraplantar injection of 1 μg Aβ_1–42_ and 2 μg SHP099. ^##^*p* < 0.01, ^###^*p* < 0.001, ****^, ####^*p* < 0.0001, two-way ANOVA with the Sidak post-hoc test; vehicle, n = 6; Aβ_1–42_, n = 10; Aβ_1–42_ + SHP099, n = 9. Data are expressed as mean ± SEM. **h:** Heat hyperalgesia in the SNI neuropathic pain model following intraplantar α_2_M and LRPAP1 (0.5 μg) injection. **i:** Heat hyperalgesia in TRPV1-cre;LRP1^flox/flox^ mice following intraplantar α_2_M injection after SNI surgery. **j:** Heat hyperalgesia assessed by the Hargreaves test in the SNI chronic pain model following intraplantar α_2_M and SHP099 (2 μg) injection. **k:** Schematic of LRP1-induced TRPV1 inhibition via SHP2 activation: The role of Aβ_1–42_ and α_2_M. Data are shown as the mean ± SEM. DRG, dorsal root ganglion; LRP1, low-density lipoprotein receptor-related protein 1; TRPV1, transient receptor potential vanilloid 1; LRPAP1, low-density lipoprotein receptor-related protein-associated protein 1; SHP2, src-homology domain-2–containing protein tyrosine phosphatase; α_2_M, α_2_-macroglobulin; cKO, conditional knockout; ANOVA, analysis of variance; SEM, standard error of the mean. (For interpretation of the references to colour in this figure legend, the reader is referred to the web version of this article.)

To understand how Aβ_1–42_ influences heat hyperalgesia through the LRP1-SHP2 pathway in the actual physiological milieu, we performed in vivo experiments. Using a capsaicin-induced acute pain mouse model generated by injecting capsaicin in the intraplantar region to activate peripheral TRPV1, we observed that Aβ_1–42_ injections significantly reduced the capsaicin-induced acute pain. However, the application of the LRP1 antagonist LRPAP1 counteracted the analgesic effects of Aβ_1–42_, suggesting that LRP1 plays a crucial role in Aβ_1–42_-mediated pain modulation (Fig. 8a).

We further examined the effect of Aβ_1–42_ in the SNI model (Fig. 8b), a widely recognized model of clinical neuropathic pain. Additionally, we created conditional knockout mice by administering AAV5-TRPV1-Cre-Tdtomato to LRP1-floxed mice, leading to the specific deletion of LRP1 in TRPV1-expressing neurons (Fig. 8c and d). In these models, the modulation of pain by Aβ_1–42_ was reversed upon administering LRPAP1 (Fig. 8e), indicating the involvement of LRP1 in chronic pain modulation. After confirming the reduced expression of LRP1 in conditional knockout mice, we observed that TRPV1-cre;LRP1^flox/flox^ mice exhibited a decreased withdrawal latency following the intraplantar Aβ_1–42_ injection (Fig. 8f). This finding underscores the essential role of LRP1 in mediating the effects of Aβ_1–42_ on heat hyperalgesia. Additionally, we explored the intermediary role of SHP2 in this process. Intraplantar injections of Aβ_1–42_ effectively reduced heat hyperalgesia in the SNI model, an effect that was reversed by the SHP2 inhibitor SHP099 (Fig. 8g). To further confirm this effect in vivo, we tested the LRP1-SHP2 pathway using α_2_M in the SNI model. Intraplantar injection of α_2_M significantly increased the PWL compared to that of the vehicle group, and this effect was reversed by LRPAP1, demonstrating that α_2_M modulates heat hyperalgesia through the LRP1-SHP2 pathway (Fig. 8h). Moreover, the α_2_M-induced reduction in heat hyperalgesia was diminished in TRPV1-cre;LRP1^flox/flox^ mice compared to controls, reinforcing the critical role of LRP1 in this process (Fig. 8i). Finally, using SHP099, we confirmed that SHP2 dephosphorylation is essential for the α_2_M-mediated inhibition of TRPV1 activity in mouse DRG neurons and the reduction of heat hyperalgesia in the SNI model (Fig. 8j). These results collectively suggest that both Aβ_1–42_ and α_2_M modulate TRPV1 activity via the dephosphorylation of SHP2.

## Discussion

Our study highlights the complex interplay between aging and alterations in pain sensitivity, revealing significantly reduced heat sensitivity in mature adults compared to younger individuals. Mature adults exhibit higher pain thresholds, indicative of distinct age-related changes in pain sensitivity. We observed a significant increase in plasma Aβ_1–42_ concentrations in mature adults compared to their younger counterparts, prompting further exploration of its association with pain perception [1,2,42]. Aβ_1–42_, which is prominently expressed in rat DRG neurons, plays a crucial role in modulating inflammatory pain and nociceptive signaling, suggesting that its elevated levels may act as a regulatory factor in the pain pathways of the PNS [4,7,16,17,43]. However, the precise mechanisms underlying Aβ_1–42_-mediated pain modulation within the PNS require further investigation.

Our findings demonstrate that Aβ_1–42_ reduces TRPV1 activation in mature adults through the LRP1-SHP2 signaling axis. This conclusion is supported by the quantitative analysis of Aβ_1–42_ concentrations across the plasma, hippocampus, DRG, and spinal cord tissues, along with mechanistic validation using Western blot and co-immunoprecipitation experiments. The specificity of SHP2 involvement was further confirmed through pharmacological studies. SHP099, a widely validated SHP2 inhibitor, effectively reversed the effects of Aβ_1–42_, while PTPiIII, a SHP1-specific inhibitor, had no impact, highlighting the distinct role of SHP2 in this pathway [44,45]. Although SHP099 is known to have off-target effects, the dose-dependent validation strengthened its specificity under the tested conditions. These findings underscore the pivotal role of SHP2 in the LRP1-mediated pathway, providing a mechanistic basis for targeting SHP2 in pain modulation.

The significance of Aβ_1–42_ extends beyond its role in nociception. While Aβ has been predominantly studied in the context of Alzheimer’s disease, where its pathological accumulation contributes to neurodegeneration and cognitive decline, emerging evidence highlights its physiological relevance in the PNS [12,46,47]. Our findings demonstrate that Aβ_1–42_ engages LRP1 to modulate TRPV1 activity, suggesting that it has both neurodegenerative and regulatory effects [18]. This duality underscores the multifunctional nature of Aβ_1–42_, which acts as a pain modulator in the PNS while contributing to plaque formation, synaptic dysfunction, and inflammation in the central nervous system [48,49].

Interestingly, α2M, another LRP1 ligand, activates LRP1 through pathways similar to those utilized by Aβ1–42, ultimately modulating nociceptive signaling. α2M, widely recognized for its roles in protease inhibition and immune regulation, has recently been shown to influence pain modulation via the LRP1-SHP2-TRPV1 axis. Our findings demonstrate that intraplantar injection of α2M increased the PWL in the SNI model, consistent with the involvement of LRP1-mediated signaling pathways similar to those activated by Aβ_1–42_ [50]. By focusing on the shared mechanisms of α2M and Aβ_1–42_, our study reveals a novel framework for understanding pain modulation through LRP1, highlighting the therapeutic potential of targeting these pathways.

The specificity of TRPV1 in thermal hyperalgesia further reinforces its critical role in nociceptive regulation. The involvement of TRPV1 in thermal and mechanical hyperalgesia is well-documented, although its functional variations across different experimental models reveal its complexity [[51], [52], [53], [54], [55], [56], [57], [58], [59]]. Here, we found that the intraplantar injection of Aβ_1–42_ mitigates heat hyperalgesia in an SNI pain model without affecting mechanical sensitivity, suggesting that it selectively modulates pain through the LRP1-SHP2-TRPV1 axis. This highlights the potential for harnessing this pathway to develop therapies for chronic pain conditions, offering targeted interventions for thermal nociception.

Our results align with prior studies demonstrating the analgesic effects of Aβ_1–42_. Song et al. [5] reported that elevated Aβ_1–42_ levels in the spinal cord dorsal horn post-chronic constriction injury (CCI) activate endogenous analgesic pathways, reducing nociceptive signaling. Similarly, we found that intraplantar injection of Aβ_1–42_ significantly mitigates heat hyperalgesia in the SNI pain model. Additionally, the practical advantages of intraplantar delivery methods—including rapid absorption, effective targeting, and minimal variability—enhance their feasibility for therapeutic applications [60].

## Conclusion

Our findings highlight the shared mechanisms of Aβ_1–42_ and α2M in activating the LRP1-SHP2-TRPV1 axis, underscoring the translational potential of this pathway for chronic pain management. By addressing both physiological and pathological roles of Aβ_1–42_, this study provides a comprehensive framework for understanding pain modulation and reveals LRP1 as a promising therapeutic target, particularly in aging-related conditions. Notably, our supplementary experiments revealed a sex-dependent effect of Aβ_1–42_, with significant reductions in heat pain sensitivity observed in male but not female mice. This finding emphasizes the complexity of Aβ_1–42_-mediated signaling and the importance of considering sex as a biological variable in future studies. Furthermore, our study demonstrates the instrumental role of the Aβ_1–42_/LRP1/SHP2 pathway in ameliorating heat hyperalgesia in the SNI pain model in the PNS. Together, these insights enrich our understanding of age- and sex-associated shifts in heat pain sensitivity and open avenues for innovative and safer neuropathic pain therapeutics targeting the Aβ_1–42_/LRP1/SHP2 axis in clinically relevant settings.

**Compliance with ethics requirement**

The study involving human dorsal root ganglion (DRG) tissues received ethical approval from the Institutional Review Board (IRB) of Samsung Medical Center (IRB number: 2018-11-063-002), with informed consent obtained from all patients. Additionally, all surgical and experimental procedures on animals were approved by the Institutional Animal Care and Use Committee (IACUC) of the College of Medicine at Gachon University in Incheon, Korea (approval number: LCDI-2020-0011, dated May 23, 2020), ensuring adherence to ethical standards for both human and animal research.

## CRediT authorship contribution statement

**Sung-Min Hwang:** Conceptualization, Writing – review & editing, Methodology, Writing – original draft, Investigation, Formal analysis. **Jueun Roh:** Writing – review & editing, Conceptualization, Formal analysis, Methodology, Investigation, Writing – original draft. **Eun Jin Go:** Writing – review & editing, Writing – original draft. **Jing-Ying Pan:** Investigation. **Jaeik Park:** Investigation. **Mahbubur Rahman:** Investigation. **YunJae Jung:** Writing – review & editing. **Sun-Ho Lee:** Writing – review & editing. **Inbo Han:** Writing – review & editing. **Gehoon Chung:** Funding acquisition, Writing – review & editing. **Sang Hoon Lee:** Writing – review & editing. **Temugin Berta:** Funding acquisition, Writing – review & editing. **Chul-Kyu Park:** Funding acquisition, Supervision. **Yong Ho Kim:** Supervision, Funding acquisition, Conceptualization.

## Declaration of competing interest

The authors declare that they have no known competing financial interests or personal relationships that could have appeared to influence the work reported in this paper.
