# Supplementary material for: The LRP1-SHP2 pathway regulates TRPV1 sensitivity in the peripheral nervous system: Insights from amyloid beta 1–42 modulation
Source: J Adv Res. 2025 Mar 5;79:461–74. doi: 10.1016/j.jare.2025.03.005 (PMC12766229; doi:10.1016/j.jare.2025.03.005)
Supplement: Supplementary Data 1 [file mmc1.pdf]

Supplementary Material

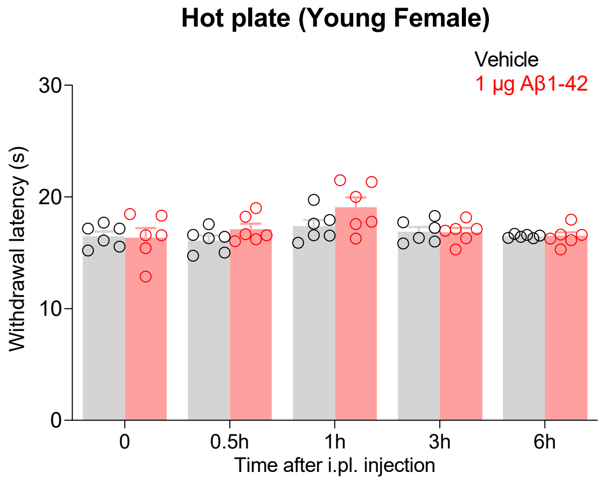

Supplemental Fig. 1. Hot plate test for basal heat sensitivity after intraplantar injection of 1 µg $A\beta_{1\text{–}42}$ in young female mice. No significant effect was observed on heat pain sensitivity, two-way ANOVA with Sidak post-hoc test, n=6/group.

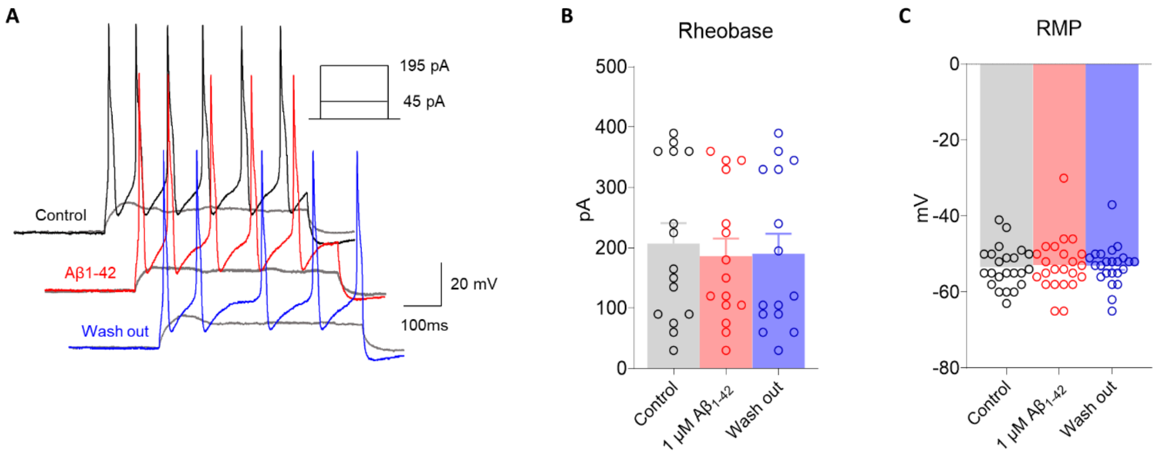

Supplemental Fig. 2. Neuronal excitability by Aβ_{1-42} treatment. (A) Whole-cell patch-clamp recordings showing action potential firing in small- and medium-sized DRG neurons during 1 μM Aβ_{1-42} treatment and washout. (B) Bar graph showing rheobase after 1 μM Aβ_{1-42} treatment and washout. One-way ANOVA, n=15/group. (C) Dot graph showing the resting membrane potential after 1 μM Aβ_{1-42} treatment and washout. One-way ANOVA, n=23/group. Data are expressed as mean ± SEM.

ANOVA, analysis of variance; DRG, dorsal root ganglion; SEM, standard error of the mean.

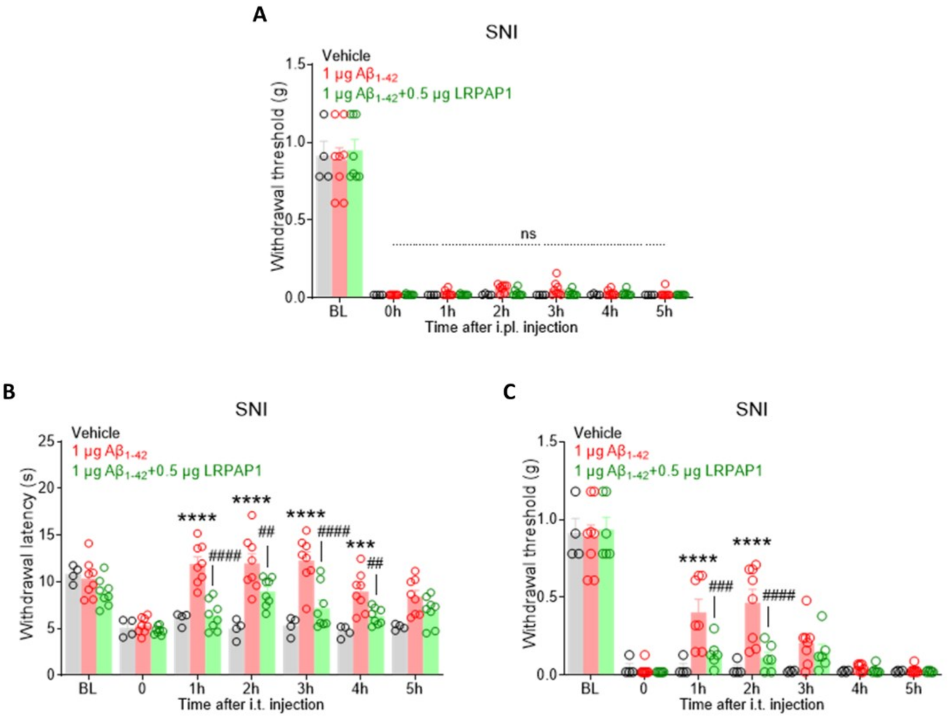

Supplemental Fig. 3. Inhibition of heat hyperalgesia and mechanical allodynia after intraplantar and intrathecal injections of A$\beta_{1\text{–}42}$. (A) Mechanical allodynia in a spared nerve injury (SNI) chronic pain model following intraplantar injection of 1 μg A$\beta_{1\text{–}42}$ and 0.5 μg LRPAP1. Two-way ANOVA with the Sidak post-hoc test; vehicle, n=4; A$\beta_{1\text{–}42}$, n=7; A$\beta_{1\text{–}42}$+LRPAP1, n=8. (B, C) Heat hyperalgesia (B) and mechanical allodynia (C) in the SNI chronic pain model following intrathecal injection of 1 μg A$\beta_{1\text{–}42}$ and 0.5 μg LRPAP1. $^{\#\#}p<0.01$, $^{***}p<0.001$, ****, $^{\#\#\#\#}p<0.0001$, two-way ANOVA with the Sidak post-hoc test. The number of mice in (B)/(C) are as follows: vehicle, n=4/4; A$\beta_{1\text{–}42}$, n=8/8; A$\beta_{1\text{–}42}$+LRPAP1, n=8/7. Data are expressed as mean ± SEM.

ANOVA, analysis of variance; LRPAP1, low-density lipoprotein receptor-related protein-associated protein 1; SEM, standard error of the mean.

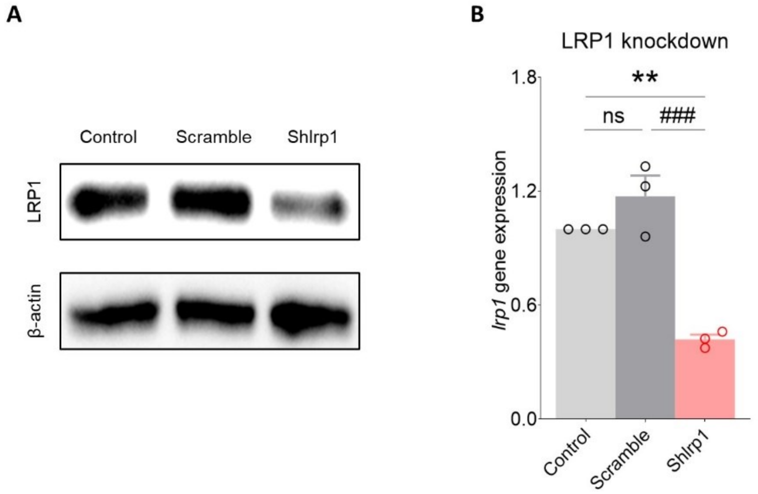

Supplemental Fig. 4. Reduction of the LRP1 gene and protein expression after lentivirus-mediated shRNA knockdown of LRP1 in a primary mouse DRG culture. (A) Bands showing LRP1 protein expression at 6 days after the transduction of scramble and shlrp1 lentivirus in mouse DRG cultures. (B) Bar graph showing normalized band intensity. ***p*<0.01, $^{###}$*p*<0.001, one-way analysis of variance, n=3.

DRG, dorsal root ganglion; ns, non-significant.
